# Depressive symptoms, parenting attitude, and violent discipline among caregivers of left-behind children in rural China: a cross-sectional study

**DOI:** 10.1186/s12889-024-18394-0

**Published:** 2024-04-09

**Authors:** Yunfei Qiu, Mengshi Li, Huifeng Shi, Chunxia Zhao, Yufeng Du, Xiaoli Wang, Jingxu Zhang

**Affiliations:** 1 Department of Maternal and Child Health, School of Public Health, Peking University, 38 Xueyuan Road, Haidian District, 100191 Beijing, PR China; 2 Department of Obstetrics and Gynecology, Peking University Third Hospital, 49 North Garden Road, Haidian District, 100191 Beijing, PR China; 3 Child Development Research Center, China Development Research Foundation, 136 Andingmenwai Street, Dongcheng District, 100010 Beijing, PR China; 4 Department of Epidemiology and Statistics, School of Public Health, Lanzhou University, 730000 Lanzhou, PR China; 5 Institute of Reproductive and Child Health/Key Laboratory of Reproductive Health, National Health Commission of the People’s Republic of China, Peking University, 100191 Beijing, PR China

**Keywords:** Depressive symptoms, Parenting attitude, Violent discipline, Left-behind children

## Abstract

**Background:**

The situation of mental health and discipline behaviors of left-behind children’s caregivers were not optimistic in rural China. Caregivers’ depression might increase the risk of using violent discipline. However, the specific ways in which depressive symptoms impact violent discipline have rarely been explored in rural areas. This study aims to assess the prevalence of violent discipline among left-behind children under 6 years of age in rural China and explore the potential mechanisms of how caregivers’ depressive symptoms affect violent discipline.

**Methods:**

We enrolled a total of 396 pairs of left-behind children and their caregivers in our study, which was conducted in 5 counties of Hebei, Henan, Jiangxi, Guizhou, and Sichuan provinces in China. The depressive symptoms of caregivers were measured by using Zung Self-rating Depression Scale (ZSDS) and violent discipline was assessed by the Child Discipline Module of Multiple Indicator Cluster Surveys (MICS). A self-designed questionnaire was utilized to measure caregiver’s parenting attitude. Based on the cross-sectional data, controlling for potential confounders, structural equation modeling (SEM) was used to assess the direct and indirect effects of the mediation models by applying the weighted least squares with mean and variance adjusted (WLSMV) estimate.

**Results:**

The prevalence of violent discipline, psychological aggression, and physical punishment was 72.7%, 59.3%, and 60.4% respectively of left-behind children under 6 years of age. According to the results of SEM, parenting attitude acted as a suppressor, suppressing the association between caregivers’ depressive symptoms and physical punishment/psychological aggression/violent discipline. The caregivers’ depressive symptoms positively influenced all the outcome variables by affecting parenting attitudes (*p* = 0.002, *p* = 0.013, *p* = 0.002).

**Conclusions:**

The presence of depressive symptoms in caregivers increases the use of violent discipline through negative parenting attitudes. The mental health status of primary caregivers of left-behind children in rural China needed emphasis and improvement.

**Supplementary Information:**

The online version contains supplementary material available at 10.1186/s12889-024-18394-0.

## Background

With the development and transformation of the economy and society, a large number of people from rural areas moved to cities in search of employment in China [1]. Due to relatively high expenses but limited income, it is difficult for migrant parents to move their whole families to cities [2]. Therefore, most of the children were left behind in their rural hometowns, and were taken care of by other family members (mostly grandparents) [3]. In our study, caregivers refer to left-behind children’s primary caregivers, including grandparents and mothers. Left-behind children are identified as those who are under 18 years old and have been left in their rural hometowns after one or both parents had relocated elsewhere for employment purposes [4]. In 2023, there were still 9.02 million children who were left behind by both parents in China, which remained a concerned issue [5].

Violent discipline refers to the use of psychological aggression or physical punishment, including a series of violent methods commonly used by many caregivers, such as spanking, verbal abuse, etc. [6]. Violent discipline towards children during their early years has detrimental effects on their overall growth and well-being. Maltreatment during childhood is associated with a reduced volume of brain regions involved in learning and memory which impacts early cognitive, motor, and emotional development [7, 8]. The negative impacts of such discipline can persist throughout their lifetime [9, 10]. However, at least half of the children worldwide have experienced disciplinary measures that rely on physical force or verbal intimidation to punish unwanted behaviors and promote desired ones [11]. In Asia, China was ranked second after India, with an estimated number of 30.2 million children aged 2 to 4 experiencing psychological aggression and 26.7 million children facing physical punishment [12]. Left-behind children were more likely to suffer from psychological aggression and physical punishment compared with non-left-behind children [13]. Approximately 73.1% of left-behind children in China reported experiencing violent discipline [14]. Currently, only a few studies have investigated the prevalence of violent discipline among left-behind children in rural areas of China [14, 15], which mainly focused on children under 4 years old.

Caregivers’ depression has been reported as a risk factor for violent discipline, but most studies mainly focused on maternal depression and were carried out among school-aged children in urban areas [16–19]. In poor regions, the situation of caregivers’ mental health is not optimistic. In Shanxi and Guizhou provinces of China, 40.3% of primary caregivers of children were depressed and the prevalence was even higher among older caregivers [20]. In China’s Qinling Mountainous region, nearly a quarter (23.6%) of primary caregivers of children reported having depressive symptoms [21]. Previous studies have discovered some mediators of the association between maternal/paternal depression and violent discipline. Marital conflict mediated the association between maternal depression and physical punishment [17]. Additionally, parental depressive symptoms impact physical punishment through parents’ negative appraisals of child behaviors [22, 23]. However, the potential mechanisms of depressive symptoms affecting violent discipline among left-behind children’s caregivers were rarely been investigated.

We hypothesized that caregivers’ depression impacts violent discipline through parenting attitudes. Parenting attitude is the overall mindset and approach when it comes to raising their children. It involves the disposition and response style that parents adopt in order to support and nurture the growth and development of their children [24]. According to the knowledge-attitude-behavior theory, a fundamental theoretical framework in public health, it posits that behavior is influenced by attitude and knowledge [25]. Therefore, we assumed that violent discipline– as a kind of parenting behavior, is affected by parenting attitudes. Parenting attitude is a complex concept and is influenced by various factors, one frequently cited psychological factor is depression [26–28]. Caregivers’ depression has a negative influence on parenting attitudes, such as showing withdrawal parenting attitudes, and less patience and tolerance toward the children [29]. Consequently, we inferred that the changed parenting attitude caused by depressive symptoms was critical to finally affecting caregivers’ discipline behaviors. Additionally, it has been demonstrated that families with lower socioeconomic status tend to believe that physical punishment is appropriate to discipline children and are less aware of the significant benefits of positive parenting behaviors [13, 30, 31].

Left-behind children may face other adverse factors that contribute to violent discipline, such as low socioeconomic status, multiple siblings within the family, both parents migrating, and caregivers having to handle heavy household work [32–34]. Under this circumstance, caregivers of left-behind children often struggle to provide them with sufficient care. Left-behind children are highly vulnerable to experiencing maltreatment when their caregivers dedicate little time and attention to them [35]. Considering all the disadvantages above, it is imperative to conduct the current study in order to enhance the development and well-being of left-behind children.

Based on the evidence above, we formulated a hypothesis that caregivers’ depressive symptoms were associated with an increased likelihood of violent discipline, and parenting attitude mediates the association. Although some existing studies focused on the violent discipline of left-behind children, few research has explored the mechanisms of how depression affects violent discipline among young left-behind children’s caregivers. Therefore, more studies are required to fill the knowledge gap. The purposes of our study are: (1) to demonstrate the current status of the violent discipline of left-behind children’s caregivers in surveyed areas of rural China; (2) to explore the relationship among caregivers’ depressive symptoms, parenting attitude, and violent discipline.

## Methods

### Participants

We used the data from the control group in the endline survey of the Rural Left-behind Children Health and Development Improvement Program (RLBCHD) in China [13, 36], which was an intervention program conducted in 5 counties of Hebei, Henan, Jiangxi, Guizhou, and Sichuan provinces from 2018 to 2020. In the baseline, one or two towns in every county were chosen as program towns, considering health facilities and socioeconomic factors. The target villages of the intervention group were selected using a multi-stage stratified sampling approach. Three villages were selected from each stratum based on the number of left-behind children under 3 years old in each town. Exceptionally, all villages were selected in Pingshan County, Hebei province due to the limited number of children under 3 years old. The villages of the control group were matched from the same county according to the number of children under 3 years old, per capita income, and distance from the county center. We finally enrolled 113 villages from 27 towns for the program.

After 2 years of home-visit intervention, left-behind children aged 24–71 months and their caregivers in the same intervention and control villages were recruited in the endline study in 2020. The exclusion criteria were as followings, children with disabilities, severe diseases, or congenital diseases. On the day of data collection, all eligible subjects in the sample villages that could be connected were investigated. Finally, a total of 396 participants from the control villages were enrolled in our study and all were valid responses. This program was approved by the Ethics Review Board of Peking University (approval number IRB00001052-17109) and all left-behind children’s caregivers provided written informed consent before data collection.

### Data collection and measurements

All the information on caregivers was collected through a face-to-face interview using a structured questionnaire by trained local health workers.

***Caregivers and family measures.*** The depressive symptoms of caregivers were measured by using Zung Self-rating Depression Scale (ZSDS) [37]. The ZSDS is a self-reported questionnaire consisting of 20 items, which has demonstrated robust reliability within the Chinese population [38]. The frequency of the symptoms described in the scale is rated from 1 (a little) to 4 (most of the time) [39]. The total score of the scale ranges from 20 to 80, with higher scores indicating more severe depressive symptoms. A standardized score (total score * 1.25) exceeding 50 is utilized as the cut-off value for defining the presence of depression [40].

The parenting attitude of caregivers was measured with a self-designed questionnaire based on the home visiting guidelines (home visiting as the intervention method). The questionnaire mainly focused on caregivers’ attitudes toward early stimulation, responsive care, and violent discipline. The caregivers were asked through a face-to-face interview, for example, the program staff would ask, “Children only cry after they are born, and communicating or interacting with them is a waste of time. Do you agree with this view?” (Parenting attitude scale shown in the supplementary material). The questionnaire is comprised of 13 items, and 1 point for each suitable answer, out of 13 points, with higher scores indicating a more favorable parenting attitude. The total Cronbach’s α coefficient for the 13 entries was 0.674, the KMO value was 0.768, the Bartlett’s spherical test χ2 = 578.587 (*df* = 66), *P* < 0.001, and the factor loadings corresponding to each entry were all > 0.5, which suggested the questionnaire provided good reliability and validity.

Violent discipline contains three indicators as followings, psychological aggression, physical punishment, and severe physical punishment. The presence of any of these three behaviors is defined as violent discipline [6]. These indicators were measured with the Child Discipline Module of Multiple Indicator Cluster Surveys (MICS) which was adapted from the Conflict Tactic Scale for Parent and Child (CTSPC) [41]. Psychological aggression encompasses behaviors such as shouting, yelling, or screaming at a child, along with the usage of offensive terms like ‘dumb’ or ‘lazy’ to call the child. Physical punishment encompasses behaviors such as shaking, hitting or slapping a child on the hand/arm/leg, hitting or spanking on the bottom or elsewhere on the body with a hard object or a bare hand, hitting or slapping on the face, head or ears, and hitting or beating hard and repeatedly. Due to the small sample size (*n* = 22) of severe physical punishment, a separate analysis of severe physical punishment was not conducted in the present study.

Caregivers’ housework stress was measured by asking them “Do you feel that there are many things you need to do around the house other than taking care of the children?”. The sociodemographic information included sex (defined based on the visible external anatomy of the newborn), age, and education level. Since the household income information provided by the respondents was highly inaccurate, the household wealth index was used to represent it– the number of electrical appliances and vehicles owned in the household: mobile phone or other network equipment, air conditioner, washing machine, refrigerator, electric rice cooker, home-used automobile/motorcycle/tricycle/electric vehicle.

***Children’s measures.*** Sociodemographic data of children were collected, including their age, sex, ethnicity, siblings, and left-behind type. The left-behind type was ascertained by asking their primary caregivers about the migration status of the mother and the father at the survey time. It was subsequently divided into two categories: children of single migrant parents and children of both migrant parents.

### Statistical analysis

The mean and standard deviation (SD) or the median and interquartile ranges (IQRs) of continuous variables and the frequency of categorical variables were evaluated in descriptive analysis. Linear regression was computed to estimate the association between the possible influencing factors and the score of parenting attitude. Adjusted odds ratios (ORs) with 95% confidential intervals (95% CIs) were computed using logistic regressions to analyze the influencing factors of psychological aggression, physical punishment, and violent discipline (as binary outcomes). The score of parenting attitude was not controlled for in the logistic regressions, since it was a mediating factor whereby caregivers’ depressive symptoms impact the violent discipline of left-behind children’s caregivers.

Next, structural equation modeling (SEM) was used to assess the direct and indirect effects of the hypothesized mediation model by applying the weighted least squares with mean and variance adjusted (WLSMV) estimate. We constructed a comprehensive model to explore the pathways between caregivers’ depressive symptoms and psychological aggression/physical punishment/violent discipline. Additionally, we examined the role of caregivers’ parenting attitude in mediating these relationships, while also taking into account any potential confounding factors identified through the regression analysis above. A good model fit is indicated by relative χ2 (χ2/*df*) < 3, comparative fit index (CFI) > 0.95, and root mean square error of approximation (RMSEA) < 0.06 [42, 43].

Descriptive and regression analyses were calculated using SPSS version 20.0 (SPSS, Inc., Chicago, IL), and SEM was conducted in Mplus (Version 7.4, Muthen & Muthen). The path graphs of SEM results were drawn using MS PowerPoint. A two-tailed *p*-value (< 0.05) was considered statistically significant.

## Results

### Characteristics of participants

Table 1 summarizes the characteristics of left-behind children and their caregivers. A total of 396 subjects were enrolled in our study. 34.6% of the participants were from Yudu County, while only 8.8% of the participants were from Pingshan County. The median age of the caregivers was 53.0 (IQR: 35.3, 58), 81.8% of them were female, and 33.1% suffered from depressive symptoms. Over half of the caregivers (54.3%) were either illiterate or had only primary school education, and merely 13.6% of the caregivers reached high school or above educational levels. Nearly 60% of the caregivers felt stressed due to the heavy housework. The median age of the children was 48.7 months (IQR: 38.9, 57.6), and the proportion of sex was relatively balanced, male and female accounted for 49% and 51% respectively. The prevalence of violent discipline was 72.7% among caregivers. 59.3% of the primary caregivers reported the use of psychological aggression and 60.4% reported physical punishment.

**Table 1:** Characteristics of left-behind children and caregivers according to violent discipline outcomes

	Violent Discipline		Total Sample
	Yes	No	
Total, n(%)	288(72.7)	108(27.3)	396
County, n (%)			
Yudu County	109 (37.9)	28 (25.9)	137 (34.6)
Lushi County	23 (8.0)	20 (18.5)	43 (10.9)
Pingshan County	22 (7.6)	13 (12.1)	35 (8.8)
Sansui County	72 (25.0)	31 (28.7)	103 (26.0)
Tongjiang County	62 (21.5)	16 (14.8)	78 (19.7)
*Child’s Characteristics*			
Child’s Age, months, Median (IQR)	48.4 (38.5∼58.2)	49.5 (39.5∼57.0)	48.7 (38.9∼57.6)
Child’s Sex, n (%)			
Male	146 (50.7)	48 (44.4)	194 (49.0)
Female	142 (49.3)	60 (55.6)	202 (51.0)
Child’s Ethnicity, n (%)			
Han	250 (86.8)	82 (75.9)	332 (83.8)
Minority	38 (13.2)	26 (24.1)	64 (16.2)
Sibling Number, Median (IQR)	1 (1∼2)	1 (0∼1)	1 (0∼2)
Left-behind Type, n (%)			
SLBC	74 (25.7)	45 (41.7)	119 (30.1)
PLBC	214 (74.3)	63 (58.3)	277 (69.9)
*Caregiver’s Characteristics*			
Caregiver’s Age, years, Median (IQR)	53.0 (45.0∼57.0)	52.0 (31.0∼58.0)	53.0 (35.3∼58.0)
Caregiver’s Sex, n (%)			
Male	50 (17.4)	22 (20.4)	72 (18.2)
Female	238 (82.6)	86 (79.6)	324 (81.8)
Caregiver’s Education Level, n (%)			
Illiteracy	36 (12.5)	10 (9.3)	46 (11.6)
Primary school	124 (43.1)	45 (41.7)	169 (42.7)
Middle school	94 (32.6)	33 (30.6)	127 (32.1)
High school or above	34 (11.8)	20 (18.5)	54 (13.6)
Housework Stress, n (%)			
No	111 (38.5)	58 (53.7)	169 (42.7)
Yes	177 (61.5)	50 (46.3)	227 (57.3)
Score of ZSDS, Mean (SD)	44.4 (10.5)	45.1 (9.1)	44.6 (10.2)
Depressive Symptom, n (%)			
Yes	95 (33.0)	36 (33.3)	131 (33.1)
No	193 (67.0)	72 (66.7)	265 (66.9)
Score of Parenting Attitude, Mean (SD)	9.4 (1.8)	10.2 (1.6)	9.6 (1.8)
Household Wealth Index, Mean (SD)	4.3 (1.2)	4.3 (1.1)	4.3 (1.1)

SD: Standard Deviation; IQR: Interquartile Range.

SLBC: Children of Single Migrant Parents; PLBC: Children of Both Migrant Parents;

ZSDS: Zung Self-rating Depression Scales.

### Caregivers’ depressive symptoms were negatively associated with parenting attitudes

Table 2 shows the results of the linear regression analysis of the parenting attitude of caregivers. The score of ZSDS exhibited a negative correlation with the score of parenting attitudes with and without adjustments (Std.est: -0.23; -0.21). Higher educational levels in caregivers indicated a better parenting attitude, significant in both adjusted and unadjusted models (Std.est: 0.24, 0.22, 0.31; 0.22, 0.20, 0.27). Housework stress inversely affected the parenting attitude (Std.est: -0.19; -0.13). Additionally, the child’s age was positively associated with the score of parenting attitude post-adjustment (Std.est: 0.10). However, variables such as the child’s ethnicity, left-behind type, and caregiver’s age were only found significant in the univariate linear regression analysis.

**Table 2:** Linear regression analysis of risk factors associated with the parenting attitude of caregivers

	Standardized univariate estimate (SE)	Standardized adjusted estimate (SE)
Score of ZSDS	-0.23 (0.01) ^**^	-0.21 (0.01) ^**^
County		
Yudu County	1.00	1.00
Lushi County	0.01 (0.30)	-0.10 (0.34)
Pingshan County	-0.07 (0.33)	-0.08 (0.35)
Sansui County	0.07 (0.23)	0.01 (0.31)
Tongjiang County	-0.24 (0.25) ^**^	-0.27 (0.28) ^**^
Child’s Age	0.04 (0.01)	0.10 (0.01) ^*^
Child’s Sex		
Male	1.00	1.00
Female	0.03 (0.18)	0.01 (0.17)
Child’s Ethnicity		
Han	1.00	1.00
Minority	0.13 (0.24) ^**^	0.03 (0.34)
Sibling Number	0.07 (0.09)	-0.05 (0.10)
Left-behind Type		
SLBC	1.00	1.00
PLBC	-0.21 (0.19) ^**^	-0.12 (0.30)
Caregiver’s Age	-0.18 (0.01) ^**^	0.01 (0.01)
Caregiver’s Sex		
Male	1.00	1.00
Female	0.03 (0.24)	0.07 (0.24)
Caregiver’s Education Level		
Illiteracy	1.00	1.00
Primary school	0.24 (0.29) ^**^	0.22 (0.29) ^**^
Middle school	0.22 (0.30) ^**^	0.20 (0.32) ^*^
High school or above	0.31 (0.35) ^**^	0.27 (0.39) ^**^
Housework Stress		
No	1.00	1.00
Yes	-0.19 (0.18) ^**^	-0.13 (0.17) ^**^
Household Wealth Index	0.09 (0.08)	0.01 (0.08)

* *p* < 0.05; ** *p* < 0.01.

Std.est: Standardized estimate; SE: Standard error; ZSDS: Zung Self-rating Depression Scales;

SLBC: Children of Single Migrant Parents; PLBC: Children of Both Migrant Parents.

### Potential influencing factors associated with violent discipline

Table 3 presents the association between psychological aggression/physical punishment/violent discipline of caregivers and the potential influencing factors. Children from minority backgrounds demonstrated a lower likelihood of experiencing psychological aggression and violent discipline (aOR: 0.31, 95% CI: 0.11∼0.76; aOR: 0.28, 95% CI: 0.08∼0.78). Similarly, children from Lushi County and Pingshan County were less likely to be physically punished and violently disciplined (aOR: 0.17, 95% CI: 0.07∼0.42; aOR: 0.30, 95% CI: 0.12∼0.74; aOR: 0.30, 95% CI: 0.12∼0.74; aOR: 0.34, 95% CI: 0.13∼0.88). Female caregivers had a greater risk of using physical punishment (aOR: 2.03, 95% CI: 1.08∼3.84). In addition, caregivers who felt stressed due to demanding housework were 2.19 times more likely to report physical punishment compared to those who did not (aOR: 2.19, 95% CI:1.40∼3.47).

**Table 3:** Multivariable logistic regression analysis of risk factors associated with psychological aggression, physical punishment, and violent discipline of caregivers

	Psychological Aggression	Physical Punishment	Violent Discipline
	aOR (95%CI)	aOR (95%CI)	aOR (95%CI)
Score of ZSDS	0.98 (0.96∼1.01)	0.99 (0.97∼1.01)	0.99 (0.97∼1.02)
County			
Yudu County	1.00	1.00	1.00
Lushi County	0.58 (0.25∼1.35)	0.17 (0.07∼0.42) ^**^	0.30 (0.12∼0.74) ^**^
Pingshan County	0.52 (0.21∼1.25)	0.30 (0.12∼0.74) ^**^	0.34 (0.13∼0.88) ^*^
Sansui County	1.85 (0.88∼4.82)	0.67 (0.29∼1.55)	1.69 (0.62∼5.49)
Tongjiang County	0.84 (0.42∼1.67)	0.55 (0.26∼1.15)	1.04 (0.46∼2.39)
Child’s Age	1.00 (0.98∼1.02)	1.00 (0.98∼1.02)	1.00 (0.98∼1.02)
Child’s Sex			
Male	1.00	1.00	1.00
Female	1.13 (0.74∼1. 73)	0.75 (0.49∼1.16)	0.73 (0.45∼1.17)
Child’s Ethnicity			
Han	1.00	1.00	1.00
Minority	0.31 (0.11∼0.76) *	0.55 (0.23∼1.31)	0.28 (0.08∼0.78) *
Child’s Sibling Number	1.20 (0.92∼1.57)	0.85 (0.65∼1.11)	1.17 (0.86∼1.60)
Left-behind Type			
SLBC	1.00	1.00	1.00
PLBC	1.73 (0.81∼3.70)	1.64 (0.75∼3.57)	2.29 (0.99∼5.30)
Caregiver’s Age	1.00 (0.97∼1.02)	1.01 (0.98∼1.04)	1.00 (0.97∼1.03)
Caregiver’s Sex			
Male	1.00	1.00	1.00
Female	1.39 (0.74∼2.58)	2.03 (1.08∼3.84) *	1.77 (0.88∼3.52)
Caregiver’s Education Level			
Illiteracy	1.00	1.00	1.00
Primary school	0.95 (0.44∼1.99)	1.30 (0.62∼2.69)	0.77 (0.31∼1.78)
Middle school	1.06 (0.45∼2.44)	2.32 (1.00∼5.43)	1.51 (0.55∼3.98)
High school or above	0.73 (0.27∼2.02)	1.71 (0.62∼4.75)	1.19 (0.38∼3.71)
Housework Stress			
No	1.00	1.00	1.00
Yes	1.49 (0.96∼2.33)	2.19 (1.40∼3.47) **	1.85 (1.13∼3.05) *
Household Wealth Index	0.92 (0.75∼1.12)	1.00 (0.81∼1.22)	0.98 (0.78∼1.23)

* *p* < 0.05; ** *p* < 0.01.

aOR: Adjusted Odds Ratio; 95% CI: 95% Confidential Interval;

ZSDS: Zung Self-rating Depression Scales;

SLBC: Children of Single Migrant Parents; PLBC: Children of Both Migrant Parents.

### Caregivers’ depressive symptoms, parenting attitude, and child abuse

**Fig. 1:**
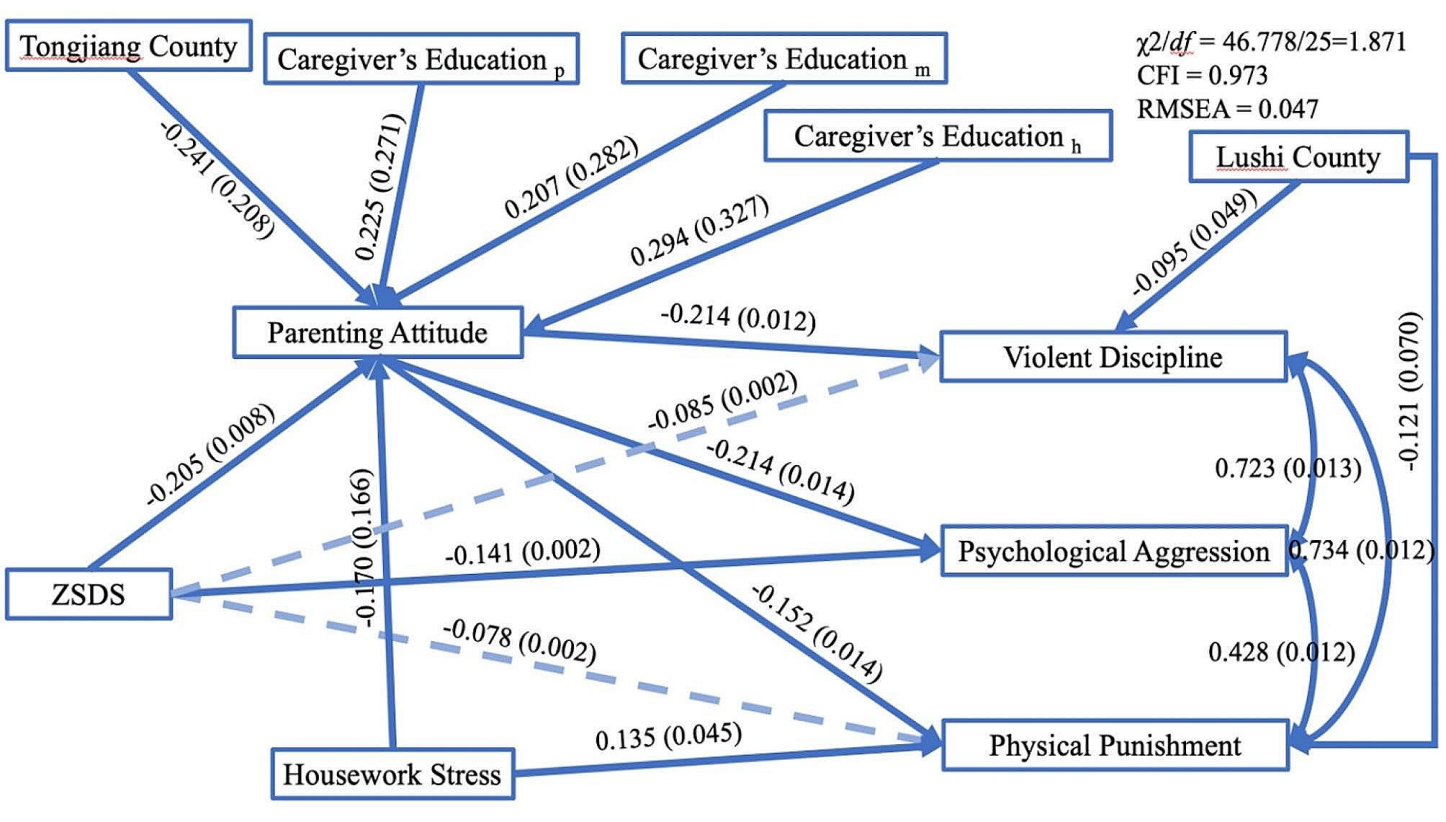
Structural equation models for explaining the relationship between psychological aggression/physical punishment/violent discipline and caregivers’ depressive symptoms (parenting attitude as mediators) after controlling for the county, caregiver’s education, and housework stress (only significant control variables were shown in the figure). Values are standardized estimated coefficients (standard error). Solid lines indicate significant associations (*p* < 0.05). Caregivers’ education _p/m/h_: caregivers were primary school, middle school, and high school or higher educated, respectively

The estimated coefficients of the structural equation models are shown in Table 4; Fig. 1. χ2/*df*, CFI, and RMSEA demonstrated that the model fitted the data well (χ2/*df* = 1.871, CFI = 0.973, RMSEA = 0.047). The results of the model showed that the overall effects of the ZSDS scores on the outcome variables were not statistically significant. Only the direct effect of the score of ZSDS on psychological aggression showed significance (Std.est. (S.E.): -0.141 (0.002), *p* = 0.005). However, the indirect effects were statistically significant and had opposite directions compared to the direct effects. For the indirect effects, the ZSDS score was negatively associated with the parenting attitude score (Std.est. (S.E.): -0.205 (0.008), *p* < 0.01), and a lower score of parenting attitude was linked to a higher prevalence of psychological aggression/physical punishment/violent discipline (Std.est. (S.E.): -0.214 (0.012), *p* < 0.01; Std.est. (S.E.): -0.152 (0.014), *p* = 0.003; Std.est. (S.E.): -0.214 (0.012), *p* < 0.01).

Psychological aggression and physical punishment (Std.est. (S.E.): 0.428 (0.012), *p* < 0.01), violent discipline and psychological aggression/physical punishment (Std.est. (S.E.): 0.723 (0.013), *p* < 0.01; Std.est. (S.E.): 0.734 (0.012), *p* < 0.01) were closely and positively related.

Based on these findings, it can be concluded that caregivers’ parenting attitudes played a role in suppressing the relationship between caregivers’ depressive symptoms and violent discipline behaviors.

**Table 4:** Standardized model estimates, standard errors, and *p*-values of hypothesized SEM models

	Effect Types	Std. est.	S.E.	Z	*p*
Path 1	Total Effect	-0.097	0.002	-1.944	0.052
	Direct Effect	**-0.141**	**0.002**	**-2.805**	**0.005**
	ZSDS→ Parenting Attitude→ Psychological Aggression	**0.044**	**0.001**	**3.089**	**0.002**
Path 2	Total Effect	-0.047	0.002	-0.960	0.337
	Direct Effect	-0.078	0.002	-1.564	0.118
	ZSDS→ Parenting Attitude→ Physical Punishment	**0.031**	**0.001**	**2.479**	**0.013**
Path 3	Total Effect	-0.041	0.002	-0.822	0.411
	Direct Effect	-0.085	0.002	-1.696	0.090
	ZSDS→ Parenting Attitude→ Violent Discipline	**0.044**	**0.001**	**3.082**	**0.002**

SEM: structural equation modeling;

ZSDS: Zung Self-rating Depression Scales;

Std. est: Standardized estimate; S.E.: Standard errors.

## Discussion

Our study showed that depressive symptoms of caregivers influenced violent discipline by affecting parenting attitudes, the total effects of these associations were suppressed by parenting attitudes. Caregivers with depressive symptoms were more likely to have negative parenting attitudes, and a lower score of caregivers’ parenting attitudes were more inclined to have violent discipline behaviors.

In the present study, the prevalence of total violent discipline was as high as 72.7%, 59.3% for psychological aggression, and 60.4% for physical punishment for left-behind children under 6 years of age. Huang Y. et al. reported a higher prevalence of violent discipline (76.4%), physical punishment (68.3%), and a similar prevalence of psychological aggression (57.5%) in rural western China [14]. Another study found similar results in rural counties of China [15]. Our research has provided additional proof regarding the widespread occurrence of violent disciplinary behaviors toward left-behind children. The issue of discipline in households with children in rural areas of China deserves more attention.

Caregivers’ parenting attitude suppressed the total effects of depression on violent discipline. Multiple studies have revealed that depression was a risk factor for violent discipline [33, 44–46]. In our research, we did not discover a significant association between the depressive symptoms of caregivers and violent disciplinary behaviors. However, we did find that caregivers’ depressive symptoms impact violent discipline through their parenting attitudes. The suppressor is characterized as an additional variable that, when included in a regression equation, enhances the regression coefficient between the independent variable and the dependent variable [47]. It offers an explanation for the presence of significant indirect effects even without a total or direct effect. In particular, opposing indirect and direct effects can obscure a total effect. In other words, the direct effect of controlling a suppressor may be greater than the overall effect if the suppressor was not controlled. In our study, the results of the SEM models showed that the directions of direct and indirect effects were opposite. Furthermore, the direct effects of controlling the parenting attitude were greater than the total effects without controlling the parenting attitude. The positive indirect effects partially offset the negative direct effects, which explained why the total effects were not significant. When the suppression effect is not controlled for, the relationship between caregivers’ depressive symptoms and violent discipline would appear to be smaller or even insignificant. Based on the definition of suppressor [48, 49], caregivers’ parenting attitude was a suppressor in these associations, suppressing the total effects of depression on violent discipline. The relationships between depressive symptoms and violent disciplinary behaviors were hidden or suppressed by the suppressor-parenting attitude. A study showed similar results that parental depressive symptoms were not direct significantly associated with physical punishment. Parents’ depressive symptoms had an indirect effect on the actual application of physical punishment, via parents’ appraisals of child behaviors [22]. In our study, we found one potential mechanism through which depression had a positive effect on violent discipline, although it was suppressed by parenting attitude. The comprehensive mechanisms underlying the impact of depression among caregivers on violent discipline are complex, with numerous other potential mediators. Further research with a larger sample size is required.

The study strongly supports evidence from previous findings that caregivers’ depressive symptoms are one of the critical risk factors for parenting attitudes [36, 50–53]. Consistent with previous studies, we found a high prevalence of caregivers’ depressive symptoms in rural China, with over one-third of caregivers of left-behind children depressed [20, 33]. Depressed caregivers reported facing greater challenges in their parenting role compared to non-depressed caregivers. Additionally, they tended to have negative attitudes such as hostility, disaffection, less sensitivity, resentment towards children, and impatience when giving instructions to guide children’s behaviors [26, 29, 54]. Previous findings have also supported that caregivers with higher education levels were more likely to provide positive parenting attitudes toward raising children [55, 56]. Housework stress was a risk factor for parenting attitudes among left-behind children’s caregivers in our study. During parental migration, left-behind children’s caregivers have to carry a heavier burden of childcare responsibilities and other housework chores. These domestic pressures contributed to negative parenting attitudes [13]. It is advisable to seek help from other family members in order to alleviate the burden of the demanding household chores.

The present study found that caregivers’ depressive symptoms increased the risk of adopting a negative parenting attitude (attitudes toward early stimulation, responsive care, and violent discipline). This negative parenting attitude was associated with a higher prevalence of all forms of violent disciplinary behaviors. Previous studies have revealed that caregivers’ attitudes toward violence against children predicted the subsequent practical application of violent behaviors [57–59]. Gretchen Antelman. et al. reported that non-violent discipline was associated with early stimulation, while violent discipline was associated with a lower likelihood of responsive care [60]. However, research regarding the impact of parenting attitudes on violent discipline is limited to date. Our investigation extends beyond this limitation by encompassing not only the caregiver’s perspective on the appropriateness of violence as a disciplinary measure but also their attitudes toward early stimulation and responsive care. Positive parenting attitudes reflect a profound understanding of child-rearing, placing more importance on the growth and development of the children. This understanding includes the awareness that using violent disciplinary methods can negatively impact early childhood development [61]. As a result, individuals with positive parenting attitudes strive to avoid such behaviors in parenting.

To our knowledge, this is the first study to explore the associations of depression, parenting attitude, and violent discipline among left-behind children’s caregivers in rural areas of China. Besides, the study provided a possible explanation of how depressive symptoms affect the violent discipline of left-behind children’s caregivers. The findings suggest that amelioration of caregivers’ mental health problems can also enhance the parenting attitudes of caregivers. It is essential to screen for depression in caregivers and implement measures to improve their mental health, parenting knowledge and attitudes in poor rural areas. The present study provides new insights for improving violent discipline and emphasizes the importance of caregivers’ mental health and parenting attitudes in raising children. Moreover, this study complemented the current status of violent discipline among left-behind children in rural China. It is imperative that greater attention be paid to their plight, and targeted interventions be developed and implemented to enhance their overall well-being.

Our study has several limitations. First, due to the cross-sectional nature of the study, the cause-and-effect relationship could not be identified. It is possible that the actual application of violent discipline aggravated caregivers’ depressive symptoms. Second, the reliability of the parenting attitude questionnaire is at a moderate level, possibly due to the limited number of entries in the questionnaire. This suggests that the scale requires further revision for enhanced consistency. Third, our sample size was restricted as the data was derived from the control group of an intervention program. Consequently, the generalizability of our findings was limited. To gain a more comprehensive understanding of these associations, it is imperative to conduct large-scale cohort studies.

## Conclusions

In conclusion, the situation concerning the depressive symptoms of caregivers of left-behind children was poor in impoverished areas of China, and the prevalence of violent discipline suffered by these children was also relatively high. Moreover, the presence of depressive symptoms in caregivers resulted in an increased risk of using violent discipline through negative parenting attitudes. Longitudinal studies are needed to further explore the causal effects in detail. Comprehensive interventions addressing caregivers’ mental health problems and spreading correct knowledge about raising children are needed to promote the well-being of left-behind children.

### Electronic supplementary material

Below is the link to the electronic supplementary material.

Supplementary Material 1

## Data Availability

The datasets generated and/or analyzed during the current study are not publicly available due to the restrictions of the local ethics committee and institutional data security and privacy policies. The data are accessible from the corresponding author (Prof Jingxu Zhang, jxzhang@bjmu.edu.cn) on reasonable request and after obtaining institutional and ethics committee’s approval.

## Abbreviations

aORs: Adjusted odds ratios
CFI: Comparative fit index
CTSPC: Conflict Tactic Scale for Parent and Child
IQRs: Interquartile ranges
MICS: Child discipline module of multiple indicator cluster surveys
ORs: Odds Ratio
95% Cis: 95% confidential intervals
PLBC: Children of Both Migrant Parents
RLBCHD: Rural Left-behind Children Health and Development Improvement Program
RMSEA: Root mean square error of approximation
SD: Standard deviation
Std.est: Standardized estimate
SE: Standard error
SLBC: Children of Single Migrant Parents
Std. coef: Standardized coefficients
SEM: Structural equation modeling
WLSMV: Weighted least squares with mean and variance adjusted estimate
ZSDS: Zung self-rating depression scale
