# Publisher Correction: Depressive symptoms, parenting attitude, and violent discipline among caregivers of left-behind children in rural China: a cross-sectional study

**DOI:** 10.1186/s12889-024-18700-w

**Published:** 2024-08-12

**Authors:** Yunfei Qiu, Mengshi Li, Huifeng Shi, Chunxia Zhao, Yufeng Du, Xiaoli Wang, Jingxu Zhang

**Affiliations:** 1 Department of Maternal and Child Health, School of Public Health, Peking University, 38 Xueyuan Road, Haidian District, Beijing, 100191 PR China; 2 Department of Obstetrics and Gynecology, Peking University Third Hospital, 49 North Garden Road, Haidian District, Beijing, 100191 PR China; 3 Child Development Research Center, China Development Research Foundation, 136 Andingmenwai Street, Dongcheng District, Beijing, 100010 PR China; 4 Department of Epidemiology and Statistics, School of Public Health, Lanzhou University, Lanzhou, 730000 PR China; 5 Institute of Reproductive and Child Health/Key Laboratory of Reproductive Health, National Health Commission of the People’s Republic of China, Peking University, Beijing, 100191 PR China

**BMC Public Health (2024) 24:994**

10.1186/s12889-024-18394-0

In the original publication of this article table 3 was not visible in the PDF due to an error in the publication process. The PDF has been rectified to amend the publication.

