# Supplementary material for: Depressive symptoms, parenting attitude, and violent discipline among caregivers of left-behind children in rural China: a cross-sectional study
Source: BMC Public Health. 2024 Apr 9;24:994. doi: 10.1186/s12889-024-18394-0 (PMC11005119; doi:10.1186/s12889-024-18394-0)
Supplement: Supplementary file 1 — Supplementary Material 1 [file 12889_2024_18394_MOESM1_ESM.docx]

**Parenting Attitude Scale**

|  | Agree | Disagree |
| --- | --- | --- |
| 1. Children only cry after they are born, and communicating or interacting with them is a waste of time  Note: “Communication and interaction” refer to playing and talking to the children |  |  |
| 2. Frequent interaction with a child through talking and playing aids in their brain development |  |  |
| 3. Singing local nursery rhymes to a child does not benefit their brain development |  |  |
| 4. Simple imitation of a child's sounds, expressions, and actions can enhance parent-child interaction |  |  |
| 5. For children aged 0-3, caregivers should promptly respond to their express needs |  |  |
| 6. The development of a child's language and intelligence is innate; external efforts are futile |  |  |
| 7. Clean and safe household items (like plastic bottles, spoons, cups, and bowls) are suitable for baby play |  |  |
| 8. Until the age of two, ensuring a child is well-fed and clothed suffices, allowing parents to work without concerns. |  |  |
| 9. When parents work away from home, it's unnecessary to prepare children for alternative care arrangements  Note: “Preparation” refers to the time spent with the alternative caregiver before the parents leaving |  |  |
| 10. Reading books and telling stories to a child should only commence once they start kindergarten |  |  |
| 11. It's inconsequential to fulfill promises made to very young children |  |  |
| 12. For 0-3-year-old children, single caregiver situations can be taxing; alternating caregiving monthly between grandmothers is a good choice |  |  |
| 13. Correcting a child's behavior effectively requires physical discipline |  |  |
